# Super-resolution *in situ* analysis of active ribosomal DNA chromatin organization in the nucleolus

**DOI:** 10.1038/s41598-020-64589-x

**Published:** 2020-05-04

**Authors:** Andreas Maiser, Stefan Dillinger, Gernot Längst, Lothar Schermelleh, Heinrich Leonhardt, Attila Németh

**Affiliations:** 1 Department of Biology II, Ludwig-Maximilians-Universität München, München, Germany; 2 Department of Biochemistry III, University of Regensburg, Regensburg, Germany; 3 Micron Advanced Bioimaging Unit, Department of Biochemistry, University of Oxford, Oxford, UK; 4 Institute of Neuropathology, Justus Liebig University, Giessen, Germany

**Keywords:** Super-resolution microscopy, Chromosomes, Nucleolus, Nucleolus, Transcription

## Abstract

Ribosomal RNA (rRNA) transcription by RNA polymerase I (Pol I) is the first key step of ribosome biogenesis. While the molecular mechanisms of rRNA transcription regulation have been elucidated in great detail, the functional organization of the multicopy rRNA gene clusters (rDNA) in the nucleolus is less well understood. Here we apply super-resolution 3D structured illumination microscopy (3D-SIM) to investigate the spatial organization of transcriptionally competent active rDNA chromatin at size scales well below the diffraction limit by optical microscopy. We identify active rDNA chromatin units exhibiting uniformly ring-shaped conformations with diameters of ~240 nm in mouse and ~170 nm in human fibroblasts, consistent with rDNA looping. The active rDNA chromatin units are clearly separated from each other and from the surrounding areas of rRNA processing. Simultaneous imaging of all active genes bound by Pol I and the architectural chromatin protein Upstream Binding Transcription Factor (UBF) reveals a random spatial orientation of regular repeats of rDNA coding sequences within the nucleoli. These observations imply rDNA looping and exclude potential formation of systematic spatial assemblies of the well-ordered repetitive arrays of transcription units. Collectively, this study uncovers key features of the 3D organization of active rDNA chromatin units and their nucleolar clusters providing a spatial framework of nucleolar chromatin organization at unprecedented detail.

## Introduction

The nucleolus is the site of the early steps of eukaryotic ribosome biogenesis, and the activity of this biological process determines its dynamic architecture^1^. Ribosome assembly initiates with the synthesis of the precursor of three rRNA molecules (18S, 5.8S, 28S) from the coding region of rRNA genes by RNA polymerase I (Pol I). The pre-rRNA undergoes multiple processing steps and gets, in part co-transcriptionally, assembled with ribosomal proteins^2^. The rRNA synthesis is a tightly regulated process that controls cell growth, and it is frequently dysregulated in disease. Highly active rRNA transcription correlates with increased tumor growth capacity, and therefore it represents also an emerging target in cancer therapy^3–5^.

Mammalian ribosomal RNA genes are separated by long intergenic spacer sequences, and their arrays form specific chromosomal domains called Nucleolar Organizer Regions (NORs). NORs are located on the short arms of the acrocentric chromosomes 13, 14, 15, 21, 22 in the human genome. The transcriptional activity and chromatin state of the repeat units vary depending on the cell’s physiological state, and variable number of active, poised, inactive and silent copies can co-exist within a single cell^6–8^. While actively transcribed rRNA genes are not detectable on silent NORs, active NORs can be interspersed with inactive genes^9^. Nucleoli form around active NORs after the mitosis and they fuse during cell cycle progression often giving rise to cells with a single nucleolus. The organization of the mammalian nucleolus has been investigated in numerous studies both *in situ* and *in vivo*. Based on the results of light and electron microscopy (EM) imaging, biochemical and molecular biology analyses, a structure-function model emerged, in which sub-nucleolar domains represent individual stages of the unidirectional process of ribosome biogenesis: The synthesis of rRNA takes place at the fibrillar center (FC) - dense fibrillar component (DFC) border, while early and late steps of ribosome maturation occur in the DFC and in the granular component (GC), respectively^10,11^. The characteristic protein components of the transcription sites and active rDNA chromatin at the FC/DFC border are the Pol I subunits and the architectural multi-HMG-box chromatin protein UBF (Upstream Binding Transcription Factor), whereas Fibrillarin (FBL) and nucleophosmin (NPM1) are marker proteins for the DFC and GC, respectively. Transcriptionally competent, UBF-bound genes constitute the nucleation sites of the cell-cycle-dependent assembly and disassembly of the nucleolus being essential determinants of nucleolar organization^8,12,13^. Despite the above findings on nucleolar organization, the spatial arrangements and interactions between active rDNA chromatin repeat units, furthermore the conformation of the individual units are poorly understood.

We address here the spatial arrangement of rDNA units at high resolution to gain understanding of the structural organization of active rDNA chromatin within the nucleolus. Our model systems, human diploid IMR90 fibroblast cells and mouse embryonic fibroblasts (MEF), represent reference human and mouse cell lines with normal diploid karyotypes. Importantly, nucleolus-associated chromosomal domains (NADs) and their dynamics under different conditions have been mapped in both model systems^14,15^, and especially for human IMR90 cells numerous functional genomics datasets are available. This provides a data-rich framework for the interpretation and integration of novel findings. We take the advantage of super-resolution multicolor 3D structured illumination microscopy (3D-SIM), which provides high-contrasted optical sectioning with 2-fold lateral and axial (8-fold volumetric) resolution increase over conventional diffraction-limited microscopy^16^, to examine the distribution and relative orientation of active rDNA chromatin units in the nucleolus. Furthermore, we show *in situ* structural organization of active rDNA chromatin at high resolution. Our novel findings excellently complement previous observations of chromosome conformation capture and EM analyses. The results reveal that active rDNA forms ring-shaped structures within mammalian nucleoli. These structures indicate looping of rDNA and complete spatial separation of each active rDNA chromatin unit. According to their size, these units likely consist of one or two transcribed rRNA genes. The UBF-bound active rDNA units are looped uniformly, that is, no linearly stretched UBF-stained nucleolar structures can be detected. In addition, looped, active units of the rDNA repeat arrays display a random rather than a specific spatial orientation in the nucleolus.

## Results

### Visualization of nucleolar organization by multicolor 3D-SIM

**Figure 1:**
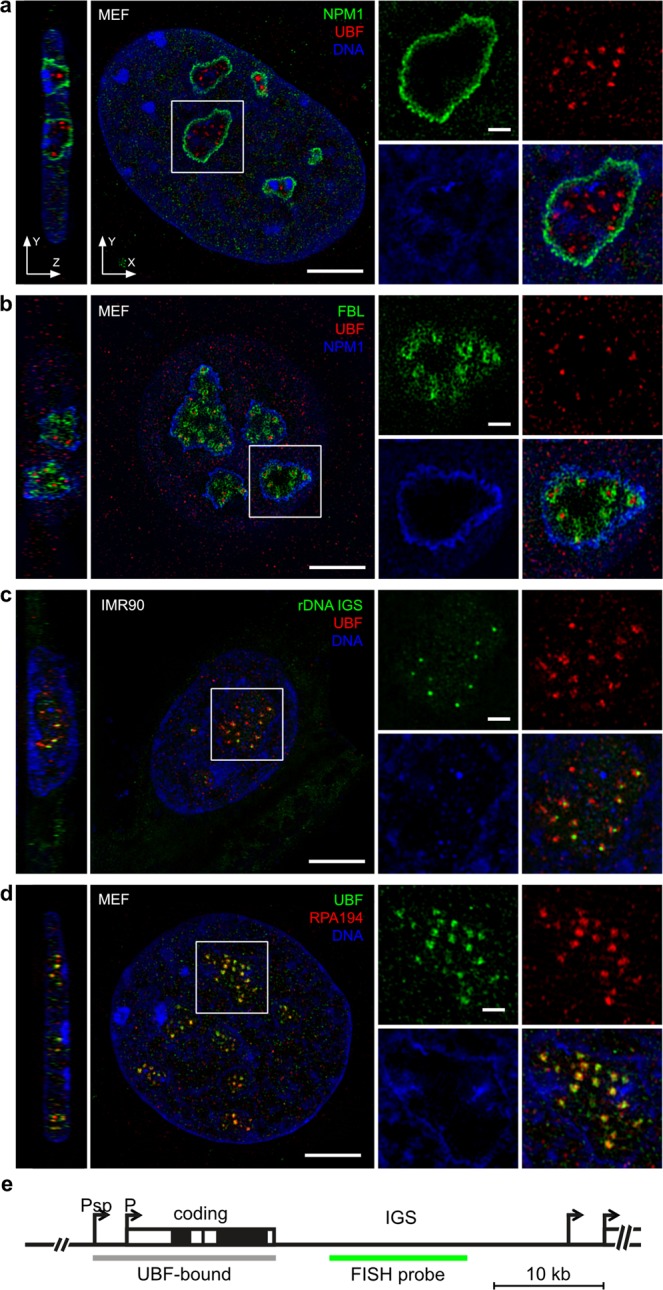
Super-resolution 3D-SIM imaging of nucleolar organization. Immunofluorescence labeling of MEF and IMR90 cells. Single nucleoli are shown in the zoom-in images. (**a**) Transcriptionally competent rRNA genes localized by UBF and the GC marker protein nucleophosmin (NPM1). (**b**) Simultaneous immunostaining of the DFC marker protein FBL with UBF and NPM1. (**c**) Immuno-FISH localization of transcriptionally active enhancer/coding rDNA (UBF) and rDNA intergenic spacer sequences (rDNA IGS). (**d**) Simultaneous immunostaining of UBF and RPA194, the largest subunit of Pol I. DNA was stained with DAPI in panels **a**, **c**, **d**. Scale bars: 5 µm on the large images and 1 µm on zoom-in images. (**e**) Schematic linear map of a single mammalian rDNA repeat unit. P_sp_: spacer promoter; P: 47S promoter; coding region: 47S rRNA gene (black brackets label 18S, 5.8S and 28S rRNA coding regions); IGS: intergenic spacer. The positions of the UBF-bound region (in active genes) and the FISH hybridization probe are shown as grey and green lines below the rDNA scheme, respectively.

To visualize active rDNA chromatin and its spatial distribution within the nucleolus, UBF immunofluorescence staining was performed in IMR90 and MEF cells in parallel with nucleophosmin staining, the marker protein for the GC. 3D-SIM imaging clearly shows that the strongest UBF signals are confined within nucleophosmin-demarcated nucleolar areas, while weaker signals can be observed also outside of this area (Fig. 1a). These observations are in good agreement with the multiple functions and nucleocytoplasmic shuttling of nucleophosmin^17^, as well as with the distinct functions of the UBF1 and UBF2 splice isoforms of UBF, which are both recognized by the UBF antibody. UBF1 is the key regulator of RNA-polymerase-I-driven rDNA transcription, whereas UBF2 was reported to possess extra-nucleolar RNA polymerase II gene regulatory function^18–20^. Next, a triple UBF/Fibrillarin/nucleophosmin staining was performed in GFP-Fibrillarin transfected cells, and imaging of the cells revealed clear separation of the early ribosome processing factor Fibrillarin from strongly stained UBF foci within nucleophosmin-marked nucleolar areas (Fig. 1b, Supplementary Fig. S1a). In order to distinguish UBF-marked enhancer and active-transcription-competent coding regions of rDNA from the rDNA intergenic spacer (IGS) sequences with super-resolution imaging, UBF and the rDNA IGS were labeled simultaneously in immuno-FISH experiments and 3D-SIM imaging was performed. Intriguingly, the resolution allows to sharply separate juxtaposed coding and labeled IGS regions (Fig. 1c,e and Supplementary Fig. S1b). However, a more precise structural analysis of the structures was hampered due to the moderate sample quality, which is possibly caused by the heat denaturation step during FISH detection. Taken together, these results provide a view of the structural organization of the mammalian nucleolus *in situ*, which clearly exceeds previous conventional multicolor fluorescence imaging approaches, and is consistent with high-resolution monochrome EM results.

### High-resolution colocalization analysis of nucleolar and extra-nucleolar UBF and Pol I

**Figure 2:**
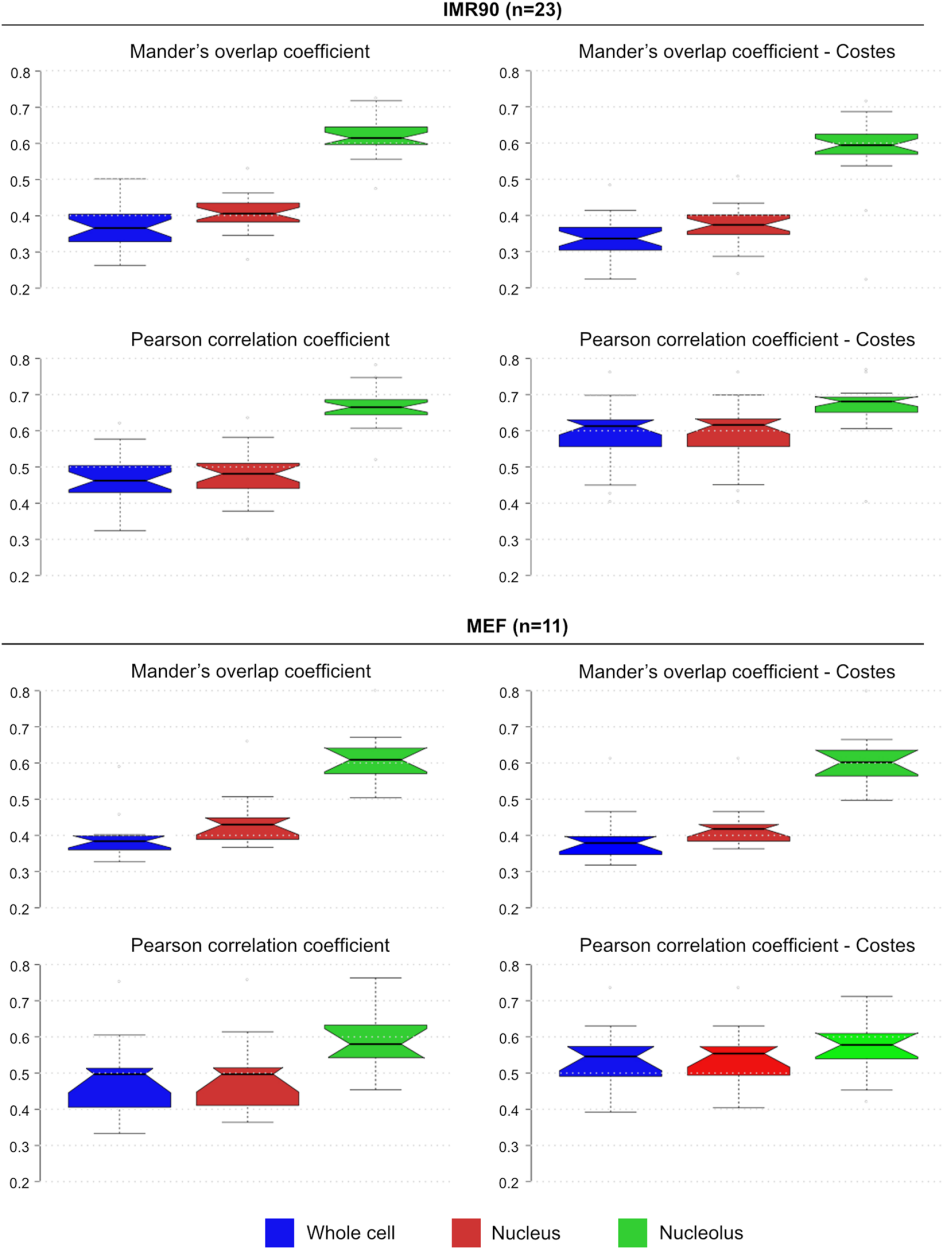
Colocalization analysis of nucleolar and extra-nucleolar UBF and Pol I. Colocalization analysis of UBF and Pol I (RPA194) signals in whole cells, nuclei and nucleoli in IMR90 (n = 23) and MEF (n = 11) cells summarized in box plots. Center lines show the medians; box limits indicate the 25th and 75th percentiles as determined by R software; whiskers extend 1.5 times the interquartile range from the 25th and 75th percentiles, outliers are represented by dots. Notches are defined as +/−1.58*IQR/sqrt(n).

To further evaluate the transcription-competent status of the UBF-marked nucleolar foci, we next performed co-staining of UBF with RPA194, the largest subunit of the rDNA-transcribing RNA polymerase I complex. The strong, clustered nucleolar UBF signals were largely overlapping with the RPA194 signals as judged by visual inspection and fluorescence intensity profiling (Fig. 1d, Supplementary Fig. S2a). The images present a visual extension of previous chromatin immunoprecipitation data on bulk rDNA. They indicate the co-binding of UBF and Pol I to active rRNA genes. A linear map of a single rDNA repeat unit (Fig. 1e) shows the position of the UBF-bound region mapped in ChIP-seq studies, which starts at the spacer promoter and finishes at the end of the coding region. To gain quantitative insight into the spatial relationship of UBF and RPA194 signals, we performed colocalization analysis in IMR90 human fibroblasts and MEF cells (Fig. 2, Supplementary Table S1). Co-occurrence analyses were performed according to Manders, Pearson’s correlation coefficients (PCC) were calculated, and the results were statistically evaluated after automatic thresholding according to Costes^21–23^. The analyses were applied on three different regions of interest (ROI): i) whole cells, ii) nuclei, iii) nucleoli. The results of colocalization analyses show that the co-occurrence of UBF and RPA194 signals, the Manders’ Overlap Coefficient (MOC), is significantly higher in the nucleolus than outside of it. Furthermore, the PCC analyses show that the overlapping UBF and RPA194 signals positively correlate in all ROI, and the highest correlation values belong to the nucleolar ROI. As Costes’ significance testing is recommended to assess the reliability of both MOC and PCC, it was applied to test also the PCC results. Interestingly, the Costes’ tests of PCC measurements show that PCC values are only marginally higher in the nucleolus than in other ROI. In summary, the combination of MOC and PCC show that the strong nucleolar UBF and RPA194 signals overlap more and their intensities correlate better than that of the overall weaker extranucleolar signals. The extranucleolar signals could represent unspecific background staining, a stained sub-population of RPA194 not engaged in rDNA transcription, UBF2 staining, or a stained UBF1 sub-population not bound to ribosomal DNA. It is worth mentioning that due to the high resolution of 3D-SIM, colocalization values are lower compared to analyses of standard widefield images, in which the signals are less well separated. However, with its 120–125 nm effective lateral resolution SIM does not reach the resolution power of single-molecule localization microscopy. RPA194 and UBF molecules reside in the nm size range and thus their colocalization analyses after immunofluorescence labeling and SIM imaging is reasonable and not substantially different from the analyses of images acquired by conventional microscopy. Altogether, the detailed analyses of UBF/RPA194 co-stained cells confirm that the UBF-labeled nucleolar structures represent active rDNA chromatin.

### UBF-marked active rDNA chromatin units form loops, which are randomly oriented in the nucleolar space

**Figure 3:**
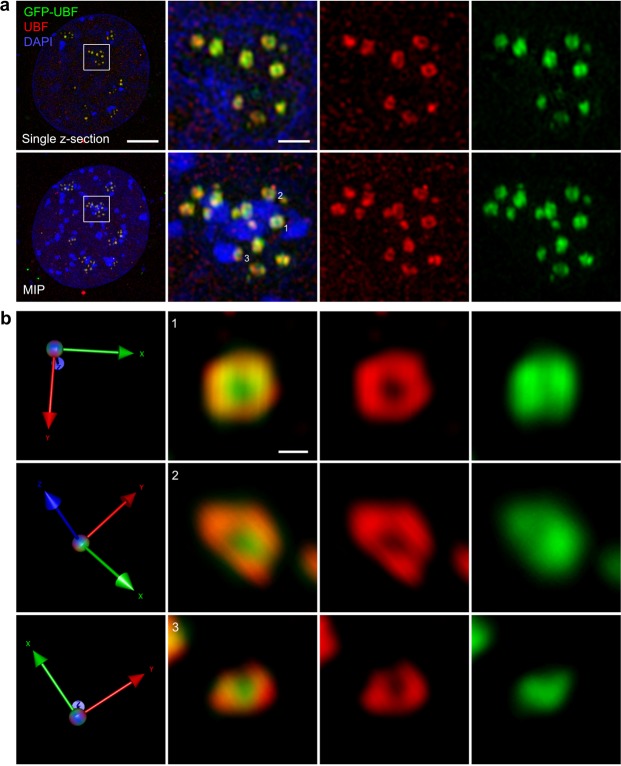
Visualization of irregularly oriented active rRNA gene loops in the nucleolus. (**a**) GFP-UBF expressing MEF cells immunofluorescent labeled with antibodies against UBF. DNA was stained with DAPI. The upper row shows a single section, the lower a maximum intensity projection (MIP) of the same nucleolus. Scale bars: 5 µm on the large image and 1 µm on the zoom-in image. (**b**) 3D volume renderings of selected UBF rings indicated in panel **a** were rotated to comparable, planar orientation with their relative XYZ orientations in the nucleolus shown on the left. Scale bar: 0.2 µm.

After validating the UBF immunofluorescence staining to study the nucleolar organization of active rDNA on high-resolution 3D-SIM images, individual UBF foci were analyzed. To further investigate specificity and also efficiency of active rRNA gene staining, MEF cells were transiently transfected with a GFP-UBF expressing plasmid DNA. The detection of recombinant UBF was enhanced by using GFP-specific fluorescent-labeled nanobodies^24^ (GFP-Booster), single-chain antibodies that due to their small size of 15 kDa ensure good epitope access in the crowded environment of the nucleolus and high labeling density. To compare the staining patterns of endogenous and recombinant UBF, cells were selected that moderately express the GFP-tagged protein and do not display strong extra-nucleolar foci of UBF, which are typical at high expression levels. Immunofluorescence staining of UBF in GFP-UBF expressing cells showed almost complete overlap indicating that endogenous UBF staining works with high quality and does not compromise high-resolution imaging of active nucleolar rRNA genes. Importantly, 3D-SIM resolved UBF foci as ring-shaped structures (Fig. 3a). Simple visual inspection of these structures suggested that the relative orientation of the rings does not display a structured, regular pattern despite the underlying tandemly repeated linear sequence arrangement of the active genes. This observation is further illustrated in magnified 3D rotated views of individual rings (Fig. 3b). Remarkably, at this resolution level some differences in the staining patterns of endogenous and GFP-tagged UBF can be observed. We speculate that the differences in the signal distributions along the ring-shaped structures could be due to competitive binding of UBF and GFP-UBF to rDNA. Another possibility is that the accessibility of the epitopes is confined and simultaneous immunofluorescence staining of endogenous and GFP-tagged UBF reduces the sensitivity of detection due to competitive binding of the antibodies. The above technical considerations could also explain why the ring-shaped structures cannot be clearly resolved when UBF and RPA194 are labeled by immunofluorescence simultaneously (Fig. 1d, Supplementary Fig. S2a). In addition, GFP-UBF signals can be seen in the middle of the rings, i.e. in the FC, while endogenous UBF is not detected here. We consider that i) some unbound UBF possibly accumulates in the FC, and the unbound/bound ratio of overexpressed GFP-UBF is higher than that of endogenous UBF, ii) due to their 10-fold smaller size GFP nanobodies may access the FC more efficiently than UBF antibodies. Importantly, similar to the endogenous UBF staining, RPA194 foci were also resolved by 3D-SIM as ring-shaped structures (Supplementary Fig. S3).

### Looped nucleolar UBF structures may represent individual active rRNA genes

**Figure 4:**
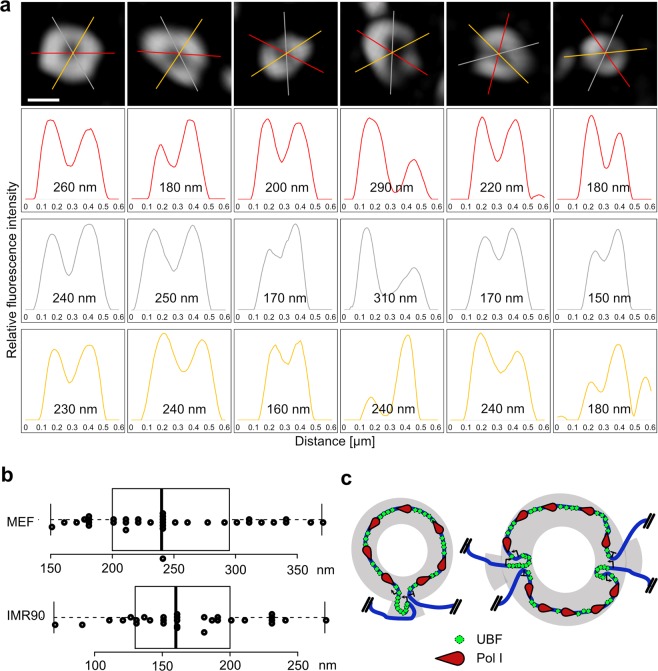
Size determination of active rRNA gene loops. (**a**) Enlarged views of individual UBF foci from MEF cells. The loops were rotated to a planar position and the diameter of each loop was determined at three positions by 60° rotation as indicated. Distances between the peaks of the fluorescence profiles are shown below the images. Scale bar: 0.2 µm. (**b**) Box plots of ring diameter measurements. Center lines show the medians; box limits indicate the 25th and 75th percentiles; whiskers extend 1.5 times the interquartile range from the 25th and 75th percentiles, outliers are represented by dots; data points are plotted as open circles. n = 36 (MEF) and n = 30 (IMR90) sample points. (**c**) Simplified hypothetic models of active rDNA chromatin loops with one or two rRNA genes per loop. Arrows label promoter and spacer promoter sites that demarcate the enhancer region, T labels the transcription termination site, red drops mark RNA polymerase I, green squares indicate UBF, and the discontinued blue line illustrates part of the IGS. The factors are not drawn to scale. The transcribed rRNA genes in the grey areas that can be correlated with the FC/DFC border represent the SIM view.

Next, we addressed the question whether single ring-shaped nucleolar UBF structures represent multi-gene transcription factories or individual transcription units separated from each other in the nucleolar space by intergenic spacer sequences. Each human and mouse rRNA repeat unit is constituted by an about 13 kb pre-rRNA coding sequence, which is preceded by a 1–2 kb long enhancer, and followed by an approximately 30–35 kb long intergenic spacer^25–27^. UBF binds *in vivo* to the enhancer and transcribed regions^28–30^, and it is associated therefore with an approximately 15 kb long sequence of an active, Pol-I-transcribed rDNA repeat unit. According to previous electron tomography measurements of Pol-I-labeled active rRNA genes in human A549 lung adenocarcinoma cells, the transcription units are confined into rather regularly sized spherical FC structures with about 270 nm in diameter^31^. We measured here the diameter of UBF rings from MEF and IMR90 cells in our 3D-SIM images. To account for the irregularities of the shape of rings, the diameter of each ring was determined by averaging fluorescence intensity peak distances in line plots at three different rotation angles (Fig. 4a, Supplementary Fig. S4). We measured a ring diameter of individual active rRNA genes of 244 ± 60 nm in MEF (n = 12) and 168 ± 47 nm in IMR90 (n = 10) cells (Fig. 4b), which is in good agreement with previous calculations from reconstructed electron tomography data^31^. We consider the following possibilities that might explain the ±20% differences in the diameter size of the rings: (i) the relative orientation of the loops to the Z-axis could account for most of the variations, as the resolution is compromised in this direction compared to XY; (ii) the loops can also be ellipsoid, not perfectly circular, and the orientation of the ellipsoid can cause superimposing effects with the Z-axis distortion; iii) differences in the transcriptional activity (Pol I loading) and thus differences in the compaction of active rDNA units may also influence the ring size. Taken together, according to the results of ring diameter measurements, the looped nucleolar UBF structures of the 3D-SIM images may represent single transcription units rather than transcription factories composed of multiple active rRNA genes. Importantly, according to this model the loop conformation requires the juxtaposition of the ends of the transcribed region, and rDNA promoter-terminator interactions are intragenic, which is depicted in a simplified illustration (Fig. 4c). However, the looped active rDNA chromatin units may contain 2–3 active rRNA genes according to a very recent study^32^. An according alternative model is also shown in Fig. 4c and discussed in more detail below.

## Discussion

We addressed in this study active rDNA organization *in situ* inside single cell nuclei at size scales down to hundred nanometers. In the past decades electron and light microscopy studies shaped our knowledge about nucleolar transcription and ribosome biogenesis. On the one side, a model with a tripartite structural organization of the mammalian nucleolus emerged. In this model the rRNA synthesis, further the early and late processing steps of the unidirectional process of ribosome biogenesis were localized to the FC/DFC, DFC and GC compartments of the mammalian nucleolus, respectively. On the other side, the introduction of the Miller spread technique^33^ enabled high-resolution electron microscopy (EM) analyses of transcribed rRNA genes. However, the nucleolar organization is disrupted in this case. Initial attempts to uncover the structural organization of rRNA transcription in the nucleolus provided valuable novel insights into the *in situ* spatial arrangement of this fundamental biological process. The structural properties of active rRNA genes have been investigated at high resolution using electron tomography imaging in a pioneering study. A four-loop model of single transcribed regions, in which each loop folds into separate coils, was proposed based on image reconstruction of fixed, Pol-I-immunogold-labeled A549 human lung adenocarcinoma cells^31^. In this EM study, the 3D arrangement of the loop was modeled based on scattered Pol I signals and the template was not visualized by UBF or rDNA staining due to technical limitations. Furthermore, the electron tomography approach required a multistep fixation and embedding protocol that could have interfered with the preservation of the 3D gene organization, and thus alternative models of the structural organization could not have been excluded. In summary, despite providing very high resolution, EM techniques are hampered by elaborate sample preparation and their inability to simultaneously and specifically label different molecules, both being overcome with fluorescence imaging approaches.

Recent super-resolution fluorescence imaging studies already uncovered some previously unknown features of nucleolar transcription. Single molecule localization microscopy revealed that FC/DFC structures are transiently disrupted during S phase, which is likely due to the interference of replication with the transcription on active rRNA genes^34^. Moreover, a dynamic, cell-cycle-dependent clustering of Pol I has been observed in quantitative live-cell super-resolution imaging experiments^35^. Nevertheless, a gap remained between imaging-based spatial models of rRNA transcription and models derived from biochemical studies. Chromosome conformation capture analyses, in which promoter-terminator interactions were demonstrated in mouse, rat and human cells^28,36,37^, strongly support a looped spatial organization model of transcriptionally competent rRNA genes. Remarkably, the ‘ribomotor’ model of yeast rRNA transcription proposed already decades ago that rDNA looping via the juxtaposition of the promoter and terminator regions might be the structural basis for enhanced RNA polymerase I transfer from termination to re-initiation^38^. Still, while the looping of the entire rDNA cluster on chromosome XII of budding yeast has been observed with different methodologies^39–41^, and looping of mammalian rDNA repeat units has also been hypothesized based on biochemical analyses^42^, detailed 3D structure analyses of individual rDNA units were lacking. Optical microscopy approaches have been successfully applied to visualize DNA loops that are very actively transcribed by RNA polymerase II on lampbrush chromosomes^43,44^. However, the looping of active rRNA or other genes on normal interphase chromosomes has not yet been observed by optical microscopy. In addition, it was not clear from chromosome conformation capture studies whether the promoter and terminator regions of the same versus different rRNA genes interact with each other, or if maybe both scenarios are possible. Notably, while the intragenic promoter-terminator interaction leads inevitably to gene looping, the intergenic interaction would be compatible both with linear and looped gene conformations. The visualization of active rDNA chromatin loops, which would fit to the size of looped single active rRNA genes argues for the possibility of the intragenic interaction. However, to test the possibility whether inter- or intragenic promoter-terminator interactions are predominant, a larger number of samples and different cell types need to be investigated in future studies.

The development of super-resolution imaging techniques continuously improves our understanding about cellular processes on the single cell level^45^. Notably, in parallel with our investigations Yao and colleagues have gained new insights into the nucleolar organization of early ribosome biogenesis by using SIM, STED and STORM approaches in live and fixed HeLa, HEK293, HFF and H9 human cells^32^. While their study focuses primarily on fibrillarin and the Dense Fibrillar Component, they have also detected ring-shaped structures in HeLa cells with endogenously tagged EGFP-RPA194 by 3D-SIM and by STED both in living and fixed cells, and with endogenous RPA194 by 3D-SIM in fixed cells. Here, by using the 3D-SIM technique we add the next piece of information to the nuclear and nucleolar perspective of rRNA transcription being able to visualize looping of active rDNA chromatin units via immunofluorescence detection of UBF and RPA194 in mouse and human cells. The 3D-SIM analyses of UBF in fixed MEFs and IMR90 cells nicely complement the results of the comprehensive study of Yao and colleagues, and the RPA194 3D-SIM images of the two studies are fairly comparable. In their model, Yao and colleagues calculate with 2–3 rRNA genes per loop based on the quantification of rDNA in all FC/DFC units by their fluorescent intensity and the number of active genes determined by quantitative PCR. Notably, the quantification of rDNA depends largely on the quality of FISH-based rDNA detection, furthermore thresholding and separation of FISH signals in the automated image analysis procedure. Thus, the calculated number of active genes might be inaccurate. Here we used a different approach to approximate the number of active genes per loop: we calculated with the diameter measurements of ring-shaped nucleolar UBF structures, and with the rDNA length and a theoretical estimation of DNA compaction. The diameter measurements of the active rDNA chromatin units allow a rough estimation of the compaction level of active rDNA. The contour length of one nucleotide is 3.3 Å (0.33 nm)^46^, and thus the UBF-marked, approximately 15 kb long enhancer and coding rDNA sequences are about 5 µm long. If we fit this into a 240 nm diameter circle with approximately 750 nm circumference, then a 6–7-fold compaction is required. Interestingly, this degree of compaction corresponds well to nucleosomal packaging, suggesting histones as potential factor for causing active rDNA compaction. Another possibility is that packaging of active rDNA is partially achieved by the architectural transcription factor UBF, which is thought to replace nucleosomes on active rRNA genes, and supposedly generates 140 bp unit enhancesomes with single 360° turns^47^. Thus, both histones and UBF, or the combination of them could account for the 6–7-fold compaction, which can be further influenced on individual genes by the activity of transcription, i.e. Pol I loading. A next level of active rDNA folding can be realized by dynamic loop formation, which might be different in individual units and vary between cell types as suggested also by our measurements: In human IMR90 cells the compaction appears to be higher than in MEF cells. Regarding this observation it should be noted that the approximately 2 kb long mouse rDNA enhancer is about three times longer than the human rDNA enhancer reference sequence^48^, and its variable size could also impact the alterations in the loop size. Also, it is more densely loaded with UBF than the coding region, which can be an additional cause for the observed larger ring size of mouse rRNA genes compared to human. Another possible explanation could be, that larger active rDNA chromatin units, especially in MEFs, contain more than one rRNA gene, as suggested by Yao and colleagues for different human cell lines. Taken together, our results and the work by Yao and colleagues are consistent with previous electron microscopy observations and open the way for the further elucidation of the spatial regulation of rRNA synthesis by super-resolution multicolor imaging. Possible alternative interpretations of the ring-shaped structures with a single gene loop and with a two genes per loop scenario are illustrated in Fig. 4c. Note that the two-gene-loop model on the right side represents only one possible arrangement. Assemblies with intragenic promoter-terminator interactions and with three genes are also imaginable. It is also unclear whether the rRNA genes of the active rDNA chromatin unit are adjacent head-to-tail arranged repeats or non-adjacent rDNA repeats on the NORs. Future investigations should resolve which structures are formed in different cell types, and whether different structures may co-exist or even undergo dynamic transition in individual cells.

## Material and Methods

### Cell culture

Mouse embryonic fibroblast (MEF) cells were cultured in Dulbecco’s modified Eagle’s medium (DMEM; Sigma) supplemented with 15% (v/v) fetal bovine serum (FBS, Biochrom), 1% non-essential amino acids (Sigma), 2 mM L-glutamine (Sigma), 1% Penicillin/Streptomycin (Sigma) and 0.1 mM ß-mercaptoethanol (Gibco). Human IMR90 fetal lung fibroblast cells were cultured in DMEM supplemented with 10% (v/v) FBS, 2 mM L-glutamine and 1% Penicillin/Streptomycin solution. Both cell lines were cultured at 37 °C with 5% CO_2_ and split at 80–90% cell confluency.

### Immunofluorescence labeling

Immunostaining was performed as described in detail^49^. Briefly, cells were grown on high precision #1,5 coverslips (Roth, LH22.1) to 80% confluency. After washing two times in PBS, cells were fixed in 2% formaldehyde in PBS for 10 min. After a stepwise exchange of fixation solution with PBS including 0.02% Tween20 (PBST) to avoid drying artifacts, cells were permeabilized with 0.5% Triton X-100 in PBST for 10 min, and then incubated for 30 min or 1 h in either 2% BSA in PBST, BlockAid (Thermo Fisher) or MAXblock (Active Motif) blocking solution to quench formaldehyde and minimize non-specific antibody binding. Primary and secondary antibodies were diluted in blocking solution and incubated each for 1 h in a dark humified chamber to prevent drying and fluorescence fading. A post-fixation step with 4% formaldehyde in PBS was performed to stabilize bound antibodies. Cells were counterstained with 1–2.5 µg/mL DAPI for 10 min, mounted in Vectashield (Vector Laboratories) and sealed with nail polish. To remove unwanted background each time six washing steps were performed before and after secondary antibody incubation and counterstaining. An additional washing step in purified water finally removed salts after counterstaining. All steps were performed at room temperature. For GFP-UBF and GFP-Fibrillarin transfection Fugene HD (Promega) and X-tremeGENE HP (Roche) was used as directed by the manufacturers. The signals of transfected cells were enhanced by applying GFP-Booster (ChromoTek) for 1 h prior to antibody immunostaining. The antibodies and other immunofluorescence reagents are listed in Supplementary Table 2.

### Fluorescence *in situ* hybridization (FISH)

3D-DNA FISH was essentially performed as described^50^ (steps 3.4.2 to 3.5(4)). Cells were grown on 12 mm diameter high precision coverslips (Roth) and washed twice with PBS before fixation with 4% formaldehyde in PBS for 10 min at room temperature. After a stepwise exchange with PBST, cells were quenched with freshly prepared 20 mM glycine in PBS for 10 min and then washed with PBST. 0.5% Triton X-100 in PBS was used for a 10 min permeabilization, before cells were washed in PBST and incubated in 20% glycerol in PBS for 1 h at room temperature. Coverslips were immersed into liquid N_2_ for approximately 6 seconds and then returned to the 20% glycerol/PBS solution. This step was repeated twice, before two washing steps in PBS for 2 min followed. The coverslips were then incubated 0.1 M HCl for 5 min. (The liquid N_2_ and HCl treatments increase the accessibility for FISH probes and antibodies.) After two times wash in PBS and then 2xSSC for 2 min each, cells were incubated in 50% formamide in 2xSSC pH 7 at 4 °C for at least 6 h. Prior hybridization the FISH probe (50 ng/µl in 50% formamide, 2xSSC pH 7, 10% dextran sulfate) was denatured for 2 min at 86 °C on a heat block. The denatured probe was subsequently incubated on ice for 2 min and 3 µl probe per Ø12 mm coverslip was placed to a glass slide. Before mounting the coverslips onto the FISH probe, excess formamide solution was removed. The coverslips were sealed with rubber cement (Fixogum) and air-dried until rubber cement has completely solidified. As a last hybridization step, the specimen was denatured for 2 min at 76 °C on a heat block, before incubating it in a 37 °C water bath at least overnight. For detection, the rubber cement was removed with the help of 2xSSC buffer and the coverslips were washed three times for 5 min with 37 °C preheated 2xSSC on a shaking platform. Additionally, the coverslips were washed three times for 5 min with 60 °C preheated 0.1xSSC under mild shaking and subsequently washed twice with 4xSSCT (4xSSC with 0.02% Tween20). Thereafter, either 2% BSA in PBST or MAXblock blocking solution was used followed by immunofluorescence labeling as described above. All detection steps were done at room temperature. UBF/Hr4-bio detection was done with antibodies listed in Supplementary Table 2. To generate the Hr4-bio hybridization probe, the pHr4 plasmid (gift of Brian McStay) containing a 12,428 bp BamHI/EcoRI fragment of the rDNA IGS (GenBank: U13369.1, nt 18,063–30,491) was labeled by nick translation using biotin-dUTP. Notably, a series of rDNA probes were not useful to assess rDNA structure by 3D-SIM as they delivered strong cross-hybridization signals that do not allow clear identification of rDNA. These included i) a human IGS/45S rDNA probe containing sequences from 5 kilobase pairs (kb) upstream of the transcription start site to 5 kb downstream of it, ii) a mouse IGS/45S rDNA probe containing sequences from 8 kb upstream of the transcription start site to 5.5 kb downstream of it, iii) the whole mouse rDNA repeat, iv) a mouse rDNA probe containing only enhancer, promoter and terminator rDNA sequences.

### Super-resolution 3D structured illumination microscopy (3D-SIM)

3D-SIM imaging was performed on a Deltavision OMX V3 microscope (GE Healthcare) equipped with a 100× 1.4 NA UPlanSApo oil immersion objective (Olympus), 405 nm, 488 nm and 593 nm diode lasers and Cascade II EMCCD cameras (Photometrics). Raw data acquisition and reconstruction was performed as described in detail^51^, using appropriate refractive index immersion oil, channel specific measured optical transfer functions (OTFs), and Wiener filter setting 0.002. Channel alignment was performed with softWoRx 6.0 and a custom-made macro in Fiji using the Multiview reconstruction, based on specific alignment sample acquisitions. Raw and reconstructed 3D-SIM image data quality was compatible with SIMcheck^52^ criteria (Supplementary Fig. S5). 32-bit reconstructed data sets were finally thresholded to discard negative values and converted to composite TIFF stacks for image analysis.

### Quantitative image analysis

UBF/RPA194 colocalization analysis was done using Volocity 6.1.2 software (Perkin Elmer). Different regions of interest (ROIs) for whole cell, nucleus and nucleoli were selected by using a rectangle or freehand shape around the different compartments. The threshold was automatically set within the software after Costes *et al*.^22^ and Pearson correlation coefficient and Mander’s overlap coefficients M1 and M2 were calculated. Box plots representing the summary statistics and the distribution of the primary data were generated by using BoxPlotR (http://shiny.chemgrid.org/boxplotr/). The notches are defined as + /−1.58*IQR/sqrt(n) and represent the 95% confidence interval for each median. Non-overlapping notches give roughly 95% confidence that two medians differ.

Fluorescence profile measurements were performed in two steps. First, RGB image stacks were loaded into Volocity and UBF ring structures were selected. These were rotated in 3D to obtain round-shaped structures in plane. After saving the new views, fluorescence intensity profiles were measured in Fiji. For each structure, intensity profiles were measured along lines of 0.6 µm or 1 µm lengths and for three angles with 60° rotation using a custom-made macro. Note, that the unequal resolution power in the Z vs XY directions accounts partially for the observed variability between the calculated diameter values of differently orientated rDNA rings.

## Supplementary information

Supplementary Information.

Supplementary Table 1.
