# Supplementary material for: Super-resolution in situ analysis of active ribosomal DNA chromatin organization in the nucleolus
Source: Sci Rep. 2020 May 4;10:7462. doi: 10.1038/s41598-020-64589-x (PMC7198602; doi:10.1038/s41598-020-64589-x)
Supplement: Supplementary file 1 — Supplementary Information. [file 41598_2020_64589_MOESM1_ESM.pdf]

SUPPLEMENTARY INFORMATION

Super-resolution *in situ* analysis of active ribosomal DNA chromatin organization in the nucleolus

Andreas Maiser[1], Stefan Dillinger[2], Gernot Längst[2], Lothar Schermelleh[3], Heinrich Leonhardt[1] & Attila Németh[2,4,*]

[1] Department of Biology II, Ludwig-Maximilians-Universität München, München, Germany

[2] Department of Biochemistry III, University of Regensburg, Regensburg, Germany

[3] Micron Advanced Bioimaging Unit, Department of Biochemistry, University of Oxford, Oxford, UK

[4] Institute of Neuropathology, Justus Liebig University, Giessen, Germany

* Correspondence and requests for materials should be addressed to A.N. (email: attila.nemeth@patho.med.uni-giessen.de)

This file contains:
Supplementary Figures S1-S5
Supplementary Table 2
Supplementary References

SUPPLEMENTARY FIGURES

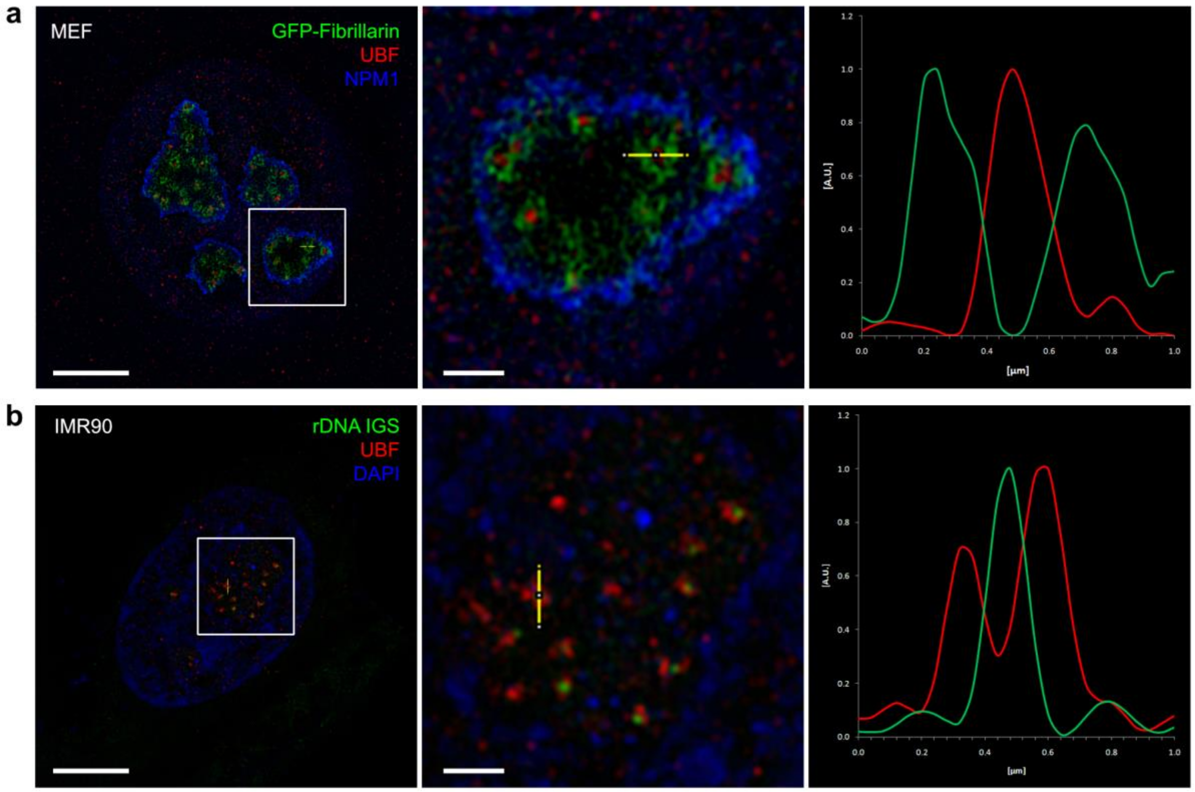

Supplementary Figure S1. Super-resolution imaging of nucleolar organization by multicolor 3D-SIM. (**a**) Simultaneous immunostaining of the FC/DFC/GC marker proteins UBF/Fibrillarin/NPM1. The fluorescence intensity profile illustrates the separation of UBF and FBL signals. (**b**) Immuno-FISH localization of transcriptionally active enhancer/coding rDNA (UBF) and rDNA intergenic spacer sequences (rDNA IGS). DNA was stained with DAPI. The fluorescence intensity profile illustrates the separation of UBF and rDNA IGS signals. Scale bars: 5 µm on the large images and 1 µm on zoom-in images.

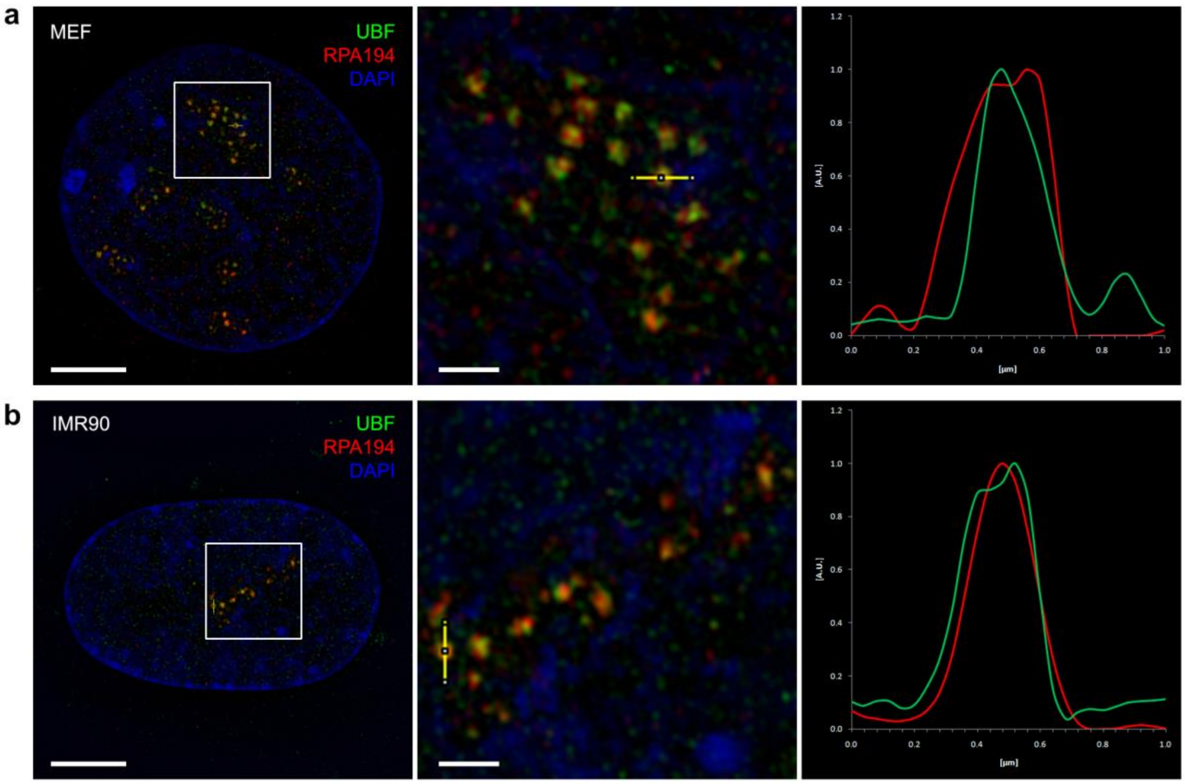

Supplementary Figure S2. Super-resolution imaging of nucleolar transcription by multicolor 3D-SIM. Simultaneous immunostaining of UBF and Pol I. The fluorescence intensity profile illustrates the colocalization of UBF and Pol I signals in (**a**) MEF cells and in (**b**) human IMR90 fibroblasts. Scale bars: 5 µm on the large images and 1 µm on zoom-in images.

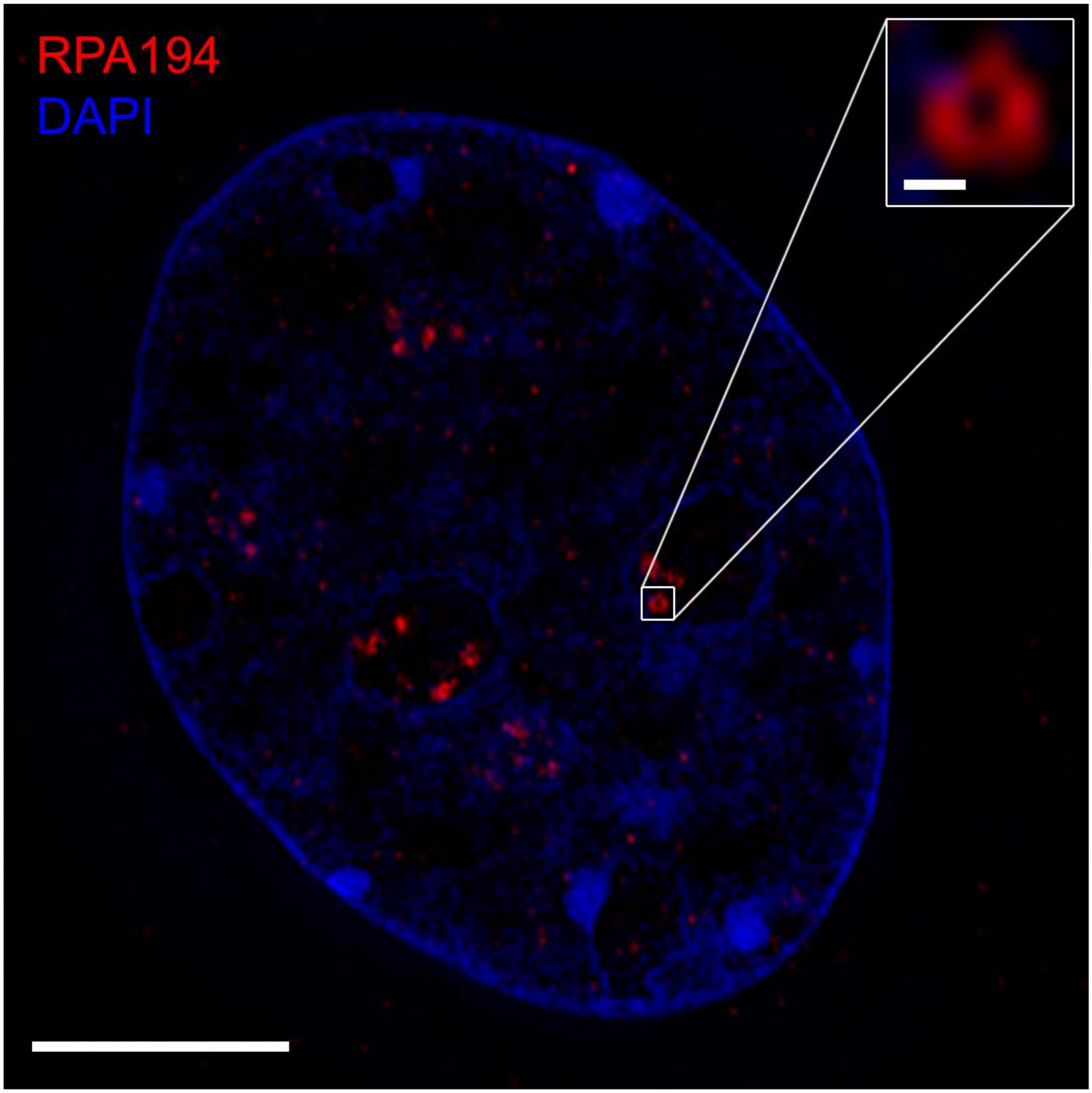

Supplementary Figure S3. Visualization of active rRNA gene loops in the nucleolus. MEF cell immunofluorescent labeled with antibodies against RPA194. DNA was stained with DAPI. A single section is shown. Scale bars: 5 µm on the large image and 0.2 µm on the zoom-in image.

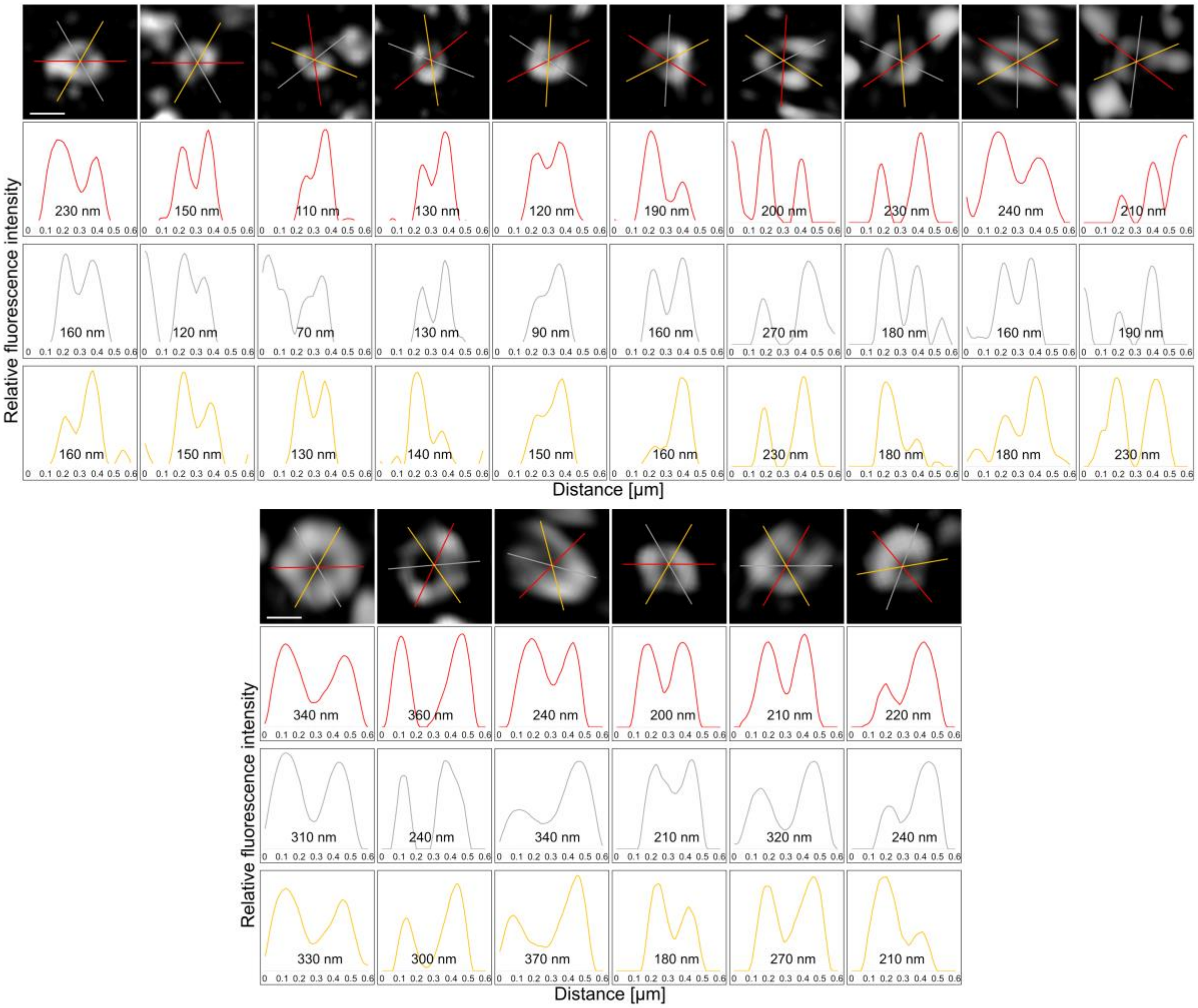

Supplementary Figure S4. Size determination of active rRNA gene loops. Enlarged views of individual UBF foci from IMR90 (upper panel, n=10) and MEF (lower panel, n=6) cells. The loops were rotated to a planar position, and the diameter of each loop was determined at three positions by 60° rotation as indicated. Distances between the relative fluorescence intensity peaks are shown below the images. Scale bar: 0.2 µm.

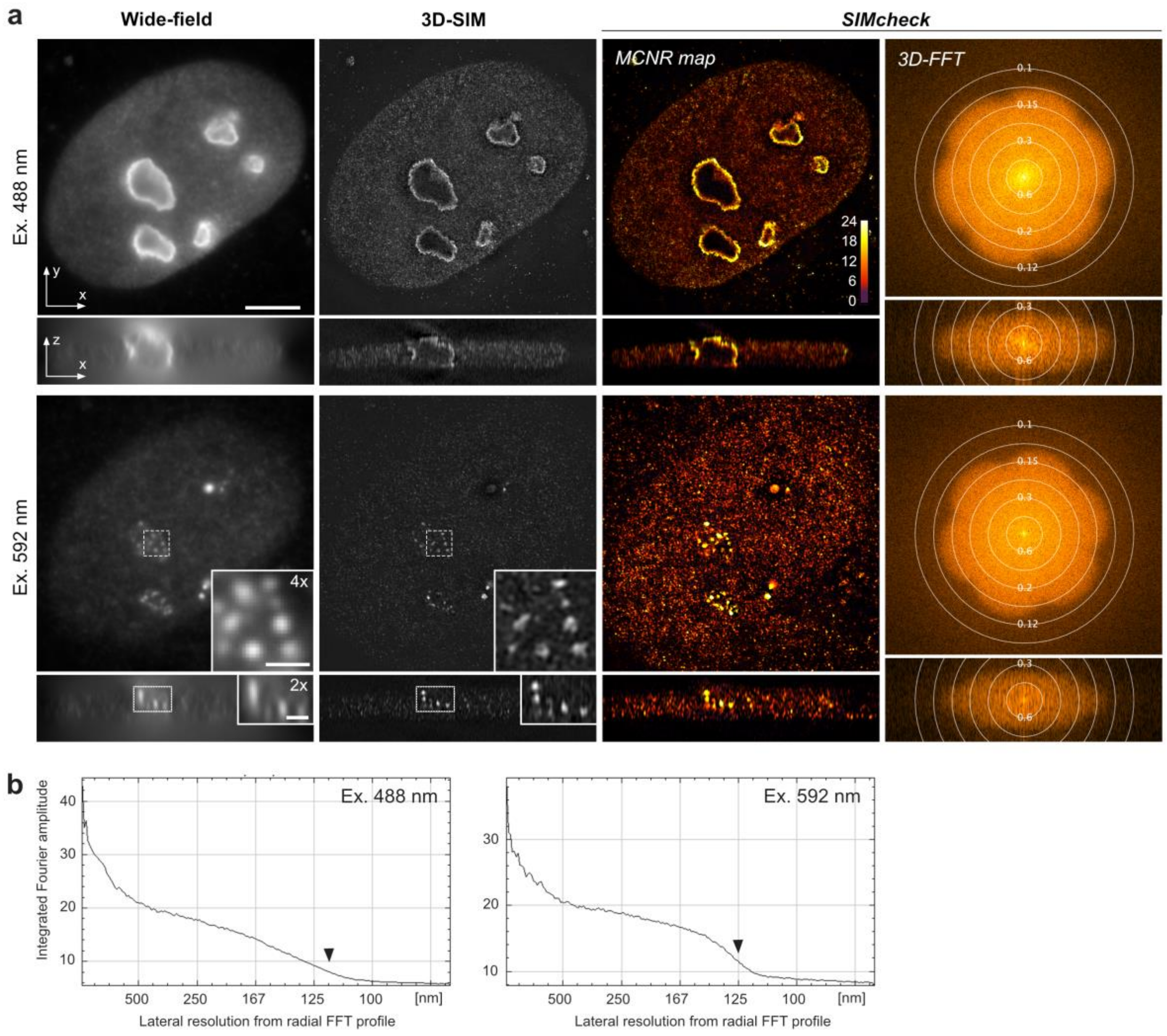

Supplementary Figure S5. *SIMcheck* analysis of 3D-SIM data quality. (**a**) First two columns: comparison between the wide-field and corresponding 3D-SIM image of the green (excitation 488 nm) and red (excitation 592 nm) fluorescence channel of the cell displayed in Fig. 1; lateral (top) and orthogonal (bottom) cross section is displayed. Column 3: corresponding quality control analysis using *SIMcheck*, showing a mapping of local stripe modulation contrast (MCNR, modulation contrast to noise ratio) on the reconstructed cross sections. Absence of purple and red color indicates high modulation contrast underlying the reconstructed structural features. Column 4: axial and lateral Fourier plots with corresponding spatial resolution indicated by concentric rings. (**b**) Averaged radial profile plots indicate effective lateral resolutions of ~120 nm in the green and ~125 nm in the red channel, respectively (arrow heads).

Supplementary Table 2. Reagents used in this study

Application	Material	Company	Cat. No
Plasmid	GFP-UBF	Addgene	17656*
Plasmid	GFP-Fibrillarin	Addgene	26673**
Blocking	MAXblock	Active Motif	15252
Blocking	BlockAid	ThermoFisher	B10710
Counterstaining	DAPI	Sigma	D9542
Immunostaining	GFP-Booster	ChromoTek	gba488
Immunostaining	B23 (mouse)	Sigma	B0556 (clone FC82291)
Immunostaining	UBF (rabbit)	Santa Cruz Biotechnology	sc-9131
Immunostaining	RPA194 (mouse)	Santa Cruz Biotechnology	sc-48385
Immunostaining	Fibrillarin (mouse)	Novus Biologicals	NB300-269 (clone 38F8)
Immunostaining	donkey anti mouse Alexa488	ThermoFisher	A21202
Immunostaining	goat anti rabbit Alexa488	ThermoFisher	A11034
Immunostaining	donkey anti mouse Alexa594	ThermoFisher	A21203
Immunostaining	donkey anti rabbit Alexa594	ThermoFisher	A21207
Plasmid (FISH)	Hr4-bio	McStay laboratory	***
3D DNA-FISH	Avidin Alexa488	ThermoFisher	A21370
3D DNA-FISH	UBF (mouse)	Santa Cruz Biotechnology	sc-13125, clone F-9

* GFP-UBF was a gift from Tom Misteli (Addgene plasmid # 17656 ; http://n2t.net/addgene:17656 ; RRID:Addgene_17656).[1]
** pEGFP-C1-Fibrillarin was a gift from Sui Huang (Addgene plasmid # 26673 ; http://n2t.net/addgene:26673 ; RRID:Addgene_26673).[2]
*** The pHr4 plasmid DNA containing the +18063/+30486 BamHI/EcoRI intergenic spacer fragment of the human rDNA (GenBank Acc. No. U13369) in pBluescript SK+ was a gift from Brian McStay.[3] The plasmid DNA was labeled with biotin-dUTP by nick translation resulting in the Hr4-bio probe for FISH analyses.

SUPPLEMENTARY REFERENCES

1. Dundr, M. *et al.* A kinetic framework for a mammalian RNA polymerase in vivo. *Science* **298,** 1623–1626 (2002).
2. Chen, D. & Huang, S. Nucleolar components involved in ribosome biogenesis cycle between the nucleolus and nucleoplasm in interphase cells. *J Cell Biol* **153,** 169–176 (2001).
3. Mais, C., Wright, J. E., Prieto, J.-L., Raggett, S. L. & McStay, B. UBF-binding site arrays form pseudo-NORs and sequester the RNA polymerase I transcription machinery. *Genes Dev* **19,** 50–64 (2005).
